# The confidence of speech-language pathology students regarding communicating with people with aphasia

**DOI:** 10.1186/1472-6920-13-92

**Published:** 2013-06-27

**Authors:** Emma Finch, Jennifer Fleming, Kyla Brown, Jennifer Lethlean, Ashley Cameron, Steven M McPhail

**Affiliations:** 1 School of Health and Rehabilitation Sciences, The University of Queensland, Brisbane, Queensland, Australia; 2 Speech Pathology Department, Princess Alexandra Hospital, Brisbane, Queensland, Australia; 3 Centre for Functioning and Health Research, Queensland Health, Brisbane, Australia; 4 Occupational Therapy Department, Princess Alexandra Hospital, Brisbane, Australia; 5 NHMRC Centre for Clinical Research Excellence, Aphasia Rehabilitation, Brisbane, Queensland, Australia; 6 School of Public Health & Social Work and Institute of Health and Biomedical Innovation, Queensland University of Technology, Brisbane, Australia

**Keywords:** Aphasia, Speech pathology, Students, Confidence, Communication, Training needs

## Abstract

**Background:**

Aphasia is an acquired language disorder that can present a significant barrier to patient involvement in healthcare decisions. Speech-language pathologists (SLPs) are viewed as experts in the field of communication. However, many SLP students do not receive practical training in techniques to communicate with people with aphasia (PWA) until they encounter PWA during clinical education placements.

**Methods:**

This study investigated the confidence and knowledge of SLP students in communicating with PWA prior to clinical placements using a customised questionnaire. Confidence in communicating with people with aphasia was assessed using a 100-point visual analogue scale. Linear, and logistic, regressions were used to examine the association between confidence and age, as well as confidence and course type (graduate-entry masters or undergraduate), respectively. Knowledge of strategies to assist communication with PWA was examined by asking respondents to list specific strategies that could assist communication with PWA.

**Results:**

SLP students were not confident with the prospect of communicating with PWA; reporting a median 29-points (inter-quartile range 17–47) on the visual analogue confidence scale. Only, four (8.2%) of respondents rated their confidence greater than 55 (out of 100). Regression analyses indicated no relationship existed between confidence and students‘ age (p = 0.31, r-squared = 0.02), or confidence and course type (p = 0.22, pseudo r-squared = 0.03). Students displayed limited knowledge about communication strategies. Thematic analysis of strategies revealed four overarching themes; Physical, Verbal Communication, Visual Information and Environmental Changes. While most students identified potential use of resources (such as images and written information), fewer students identified strategies to alter their verbal communication (such as reduced speech rate).

**Conclusions:**

SLP students who had received aphasia related theoretical coursework, but not commenced clinical placements with PWA, were not confident in their ability to communicate with PWA. Students may benefit from an educational intervention or curriculum modification to incorporate practical training in effective strategies to communicate with PWA, before they encounter PWA in clinical settings. Ensuring students have confidence and knowledge of potential communication strategies to assist communication with PWA may allow them to focus their learning experiences in more specific clinical domains, such as clinical reasoning, rather than building foundation interpersonal communication skills.

## Background

Aphasia is an acquired language disorder resulting from damage to the brain; typically as a result of cerebrovascular accident or other neurological injury. Although figures vary internationally, studies on the incidence of aphasia suggest that approximately one quarter to one third of all individuals admitted to hospital with acute stroke will present with aphasia [1-4]. Aphasia creates a substantial barrier to communication, frequently leading to social isolation and an inability to discuss or negotiate issues related to daily life including healthcare [5-9].

Previous research suggests that only 1.5 to 7.6% of the general population has basic knowledge about aphasia [10]. Kagan [11] reported that many people are often unaware of the cognitive and social competence of people with aphasia (PWA) and as a consequence avoid conversations with PWA. This can result in the exclusion of PWA from decisions about daily life and healthcare, and can have detrimental effects on the psychosocial wellbeing and quality of life of PWA [5,6,11-13]. Involving PWA in their treatment planning can lead to increased patient motivation, increased effectiveness of health professional time use, and the achievement of more holistic management programs [14]. Equipping health professionals with the skills and confidence to communicate effectively with PWA is, therefore, an important step towards optimizing patient involvement in rehabilitation.

In the healthcare setting, language barriers often prevent PWA from being involved in the design of their treatment programs or identification of rehabilitation goals [14]. PWA may also be unable to ask questions about their medical condition or treatment [5]. Evidence from an observational study in an acute stroke unit suggests that health professionals’ level of knowledge, communication skills and attitudes can act as barriers to effective communication with individuals with aphasia in hospital [15]. Knight et al. [16] found that when communicating with stroke patients without aphasia, 22% of health professionals‘ time was devoted to the dissemination of health information; however, when communicating with stroke patients with aphasia, only 7% of the health professionals’ time was spent on information dissemination. It was also found that health information was only provided to people with aphasia when a significant other was present [16]. Two possible reasons for this observation proposed by the researchers were that health professionals may have reduced confidence when communicating with PWA, or secondly that many health professionals may not be aware of the competence and value of communicating with PWA [16]. Patient-centred or client-centred care is now widely recognised as a foundation principle of appropriate healthcare and there is evidence that increased patient involvement in rehabilitation leads to better outcomes. The studies by Leach et al. [14], Knight et al. [16], and O’Halloran et al. [15] all included speech-language pathologists (SLPs) in their health professional cohorts suggesting that communicating with PWA may be problematic even amongst SLPs. However the extent of the problem has not been investigated specifically for SLPs or SLP students.

Speech and language pathologists are viewed as communication experts in hospital settings. However, while all students receive theoretical foundation knowledge about communicating with PWA through academic coursework, not all SLP students receive practical training during their coursework lectures in techniques to communicate with PWA prior to clinical education placements (where practical training refers to hands on experience communicating with PWA). The combination of the pressure of a novel clinical environment and unfamiliarity with the practical application of strategies to effectively communicate with PWA has the potential to create anxiety for novice students. In other health disciplines including medicine and nursing, clinical educators and students have reported a lack of preparedness to cope with the basic communication and interaction requirements inherent in client contact [17]. This interfered with students’ abilities to maximise learning during clinical education placements, and took the focus off student development in other domains such as goal-setting and clinical reasoning [17]. Anxiety surrounding basic communication with clients may be even more critical for SLP students as they are interacting with individuals with a communication disability, so their interpersonal skills and clinical skills are closely interrelated. Indeed, qualitative research by Jagoe and Roseingrave [18] suggested that SLP students may experience considerable apprehension at the prospect of communicating with PWA. During the study by Jagoe and Roseingrave [18] the students wrote reflective letters to themselves at the start and completion of a service learning module, which involved pairs of students visiting a PWA. Data analysis involved thematic analysis of the letters. However, out of the cohort of 22 students who participated in the module, only eight students consented to their letters being used for the study, with only six of these students providing both pre and post letters [18]. As a result, further research involving a larger student sample and a direct self-reported confidence rating is required to investigate the confidence of SLP students when communicating with PWA.

Although all SLP students are required to have achieved specific competencies for entry level practice prior to graduation, they may have substantially varying levels of confidence and experience interacting with PWA as aphasia-related clinical education placements frequently involve inequitable and arbitrary clinical experiences for students for a number of reasons, including variation in type and severity of clients’ communication disabilities and attendance rates [17]. This is particularly pertinent given that repeated experience in clinical skills has been linked to improved knowledge and confidence with the skills [19,20]. Effective communication with clients is featured as an underlying theme in many of the entry requirement areas ranging from assessment, treatment planning to treatment evaluation nationally in Speech Pathology Australia’s Competency-based Occupational Standards for Speech Pathologists Entry Level (CBOS) [21] and internationally in the American Speech-Language-Hearing Association’s Standards and Implementation Procedures for the Certificate of Clinical Competence in Speech-Language Pathology [22]. Following graduation SLPs do not usually receive further formal training in practical techniques to communicate effectively with PWA and must rely on a “learning on the job” approach to gain skills. While confidence and skills in communicating with PWA may be achieved with time and experience, it would be preferential for graduating SLPs to already have confidence and knowledge of strategies that may be implemented to assist communication with people with PWA. This is particularly important given that other health professionals will likely look to them for guidance in the field of communication when working with clients with aphasia. It remains unknown whether entry level coursework on the nature and treatment of aphasia prepares SLP students sufficiently to communicate effectively with PWA prior to their first clinical education placements with PWA. It is likely that confidence and knowledge of a range of potential communication strategies to assist in basic communication with PWA might alleviate the students’ focus on developing foundation level interpersonal communication during clinical placements and allow the students to instead maximize their learning experiences in more specific clinical domains; such as clinical reasoning.

An important first step is to investigate students’ confidence levels and their knowledge of strategies to assist communication with PWA after these students have participated in routine academic coursework about aphasia. This information could then be used to justify (or refute the need for) subsequent development and evaluation of tailored practical skills programs that could be provided as an adjunct to existing theoretical based academic courses. Consequently, the present study aimed to investigate SLP students’ self-reported confidence levels in communicating with PWA and their knowledge of strategies that could be used to assist communication with PWA.

## Methods

### Design

A cross-sectional study using a customised questionnaire was conducted with a single cohort of SLP university students.

### Participants and setting

Participants were a convenience sample of students completing an entry level SLP degree recruited from a single university site. The students were either undergraduate students or graduate entry masters students. Students in these two SLP entry level degrees complete comparable coursework. The graduate entry master’s program requires students to complete pre-requisite subjects comparable to 1st year undergraduate SLP subjects prior to enrolment in the graduate entry masters program. The graduate entry masters program then utilizes a ‘summer semester’ to enable three semesters of coursework to be completed within each calendar year (whereas undergraduate students complete two semesters and then have a long summer break). So while students’ from the undergraduate and postgraduate entry level programs were included in the sample, they were all at a comparable stage in their entry level training; which covers the same SLP related coursework.

Students from these entry level programs that were enrolled in the acquired adult neurogenic language disorders subject were invited to participate. A total of 126 students were enrolled in the subject, and all students were invited to complete the voluntary survey; 49 (38.9%) students consented to participate in this investigation. This subject contained a compulsory aphasia coursework component, which was attended by all students. The aphasia component of the subject consisted of 12 × 2 hour lectures (24 hours in total) focusing on theory including the assessment, differential diagnosis, clinical management (including goal setting, acute management and the World Health Organisation’s International Classification of Functioning, Disability and Health model (WHO) [23]; rather than practical interactions with individuals with acquired language disorders. As part of this coursework, the students received a single lecture by an individual with chronic mild aphasia. Therefore, participating students had attended lectures about aphasia as part of their routine academic coursework, but had not participated in clinical education placements with PWA. Students were selected from this point in their entry level education program to ensure they had appropriate theoretical understanding of aphasia, but not real life clinical practice experience. There were no other inclusion or exclusion criteria. At the Australian university setting for this investigation, students do receive clinical exposure as part of their academic coursework. However, the students participating in this investigation had not yet completed clinical placements in the clinical situations that expose students to PWA.

### Questionnaire

**Figure 1:**
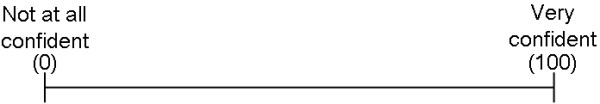
The 100 mm visual analogue scale for rating confidence in communicating with people with aphasia; scale anchors were “Not confident at all (0)” and “Very confident (100)”.

A self-report questionnaire with three short sections was developed for the purposes of the study based on information from Connect – The Communication Disability Network in the United Kingdom. The first section recorded demographic and background information (gender, age, degree enrolment and prior contact with PWA). The second section asked participants to rate their confidence in communicating with PWA by marking (drawing) a vertical line on a horizontal 100 mm visual analogue rating scale (Figure 1) with anchors of *Not at all confident (0)* through to *Very confident (100)*. The third section was an open ended question that asked participants to: *Please describe specific strategies that could be used in clinical settings to assist communication between health professional and PWA.*

### Procedure

Ethical approval for the study was obtained from the Metro South Health Service District Human Research Ethics Committee and The University of Queensland Medical Research Ethics Committee. The students were recruited during a brief presentation about the study by one member of the research team at the end of one of their aphasia lectures (to minimise any potential conflict of interest the member of the research team was not involved in the coordination or teaching of the subject). The students were informed that participation in the study was voluntary, that it would have no influence upon their subject grade, and did not contribute towards course credit. Any student who was interested in participating in the study was given the questionnaire, study information sheet and consent form by the research team member. Written informed consent was obtained from all participants prior to their participation. Consenting students completed the self-report questionnaire and returned the questionnaire to a member of the research team.

### Analysis

**Figure 2:**
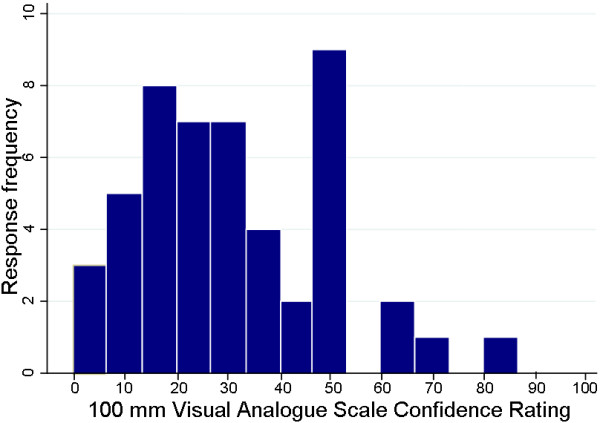
Frequency histogram of student (n = 49) visual analogue scale confidence ratings.

Quantitative analysis was undertaken using Stata IC and SPSS software packages. Demographic information was described using conventional descriptive statistics. To investigate whether enrolment in the undergraduate versus graduate entry masters program was associated with confidence ratings, a logistic regression was undertaken. Similarly, a linear regression was undertaken to investigate whether an association between the age of participants and their confidence rating existed. Confidence rating visual analogue scale data were described using median and inter-quartile range (IQR). Additionally a frequency histogram was used to examine the distribution of the confidence rating scale responses (Figure 2). Thematic analysis was used to describe the nature of qualitative written responses for potential strategies that could be used when communicating with PWA [24-28]. For the thematic analysis, two members of the research team independently coded each phrase listed by the students under emerging response categories (e.g., “slow down rate” and ”reduce rate” were both coded into the response category “reduced rate of speech”). These response categories were then aligned under emerging themes (e.g., “reduced rate of speech” was aligned under “verbal communication strategies’). A third independent researcher was available to arbitrate any unresolved disagreement between the two primary coders, but was not required (as no unresolved discrepancy between the first two coders occurred). The number of potential strategies identified by each respondent was also recorded. Additionally, the number of coded phrases in each response category was summed as a gross indicator of the most prominent strategies or resources reported by the students. In addition to describing the nature of the listed strategies, two experienced SLP members of the research team classified each strategy into one of two suitability categories (Table 1 and Table 2). Strategies considered likely to be suitable based on previous literature [25,29-33] and the coders’ professional experience are displayed in Table 1. The remaining response strategies were judged to be of uncertain benefit or insufficiently described to determine potential suitability by the experienced SLP team members and were listed separately (Table 2). For example, a listed strategy of “technology” was not considered a clear strategy description and was therefore assigned to Table 2. However, a description of using “images via an ipad” would have been considered to be a potentially suitable strategy and would have been allocated to Table 1.

**Table 1:** Thematic analysis of potential strategies suggested by the students for communicating with PWA, and the number of times (n) strategies were coded to each response category

**Themes**	**Response categories**	**n**	**%**
Physical strategies	Use of gesture	21	14.4
	Facial expressions	1	0.7
	Eye contact	2	1.4
	Face the speaker	1	0.7
Verbal communication strategies	Reduced rate of speech	18	12.3
	Short/clear sentences	9	6.2
	Simple/concise language	5	3.4
	Y/N or forced choice questions	1	0.7
	Allow the PWA time to respond	4	2.7
	Be patient	3	2.1
	Reassurance that we know they have something to say	2	1.0
	Rephrasing	1	0.7
	Clarifying	1	0.7
	Convey one idea at a time	1	0.7
Visual information	Writing things down/key words	25	17.1
	Use of images (including photographs and diagrams)	21	14.4
	Aphasia friendly written materials	10	6.9
	Word boards, picture boards or number boards	9	6.2
	Draw	1	0.7
Environmental changes	Reduce background noise	1	0.7
	Family/Friends	2	1.4

**Table 2:** Strategies of uncertain benefit or insufficiently described to determine potential suitability

**Response type**	**Response**
General therapy	Colour coding
techniques	Numbering
	Collaborative goal setting
	Functional therapy
	Visual models
	Speak as naturally as possible
Other verbal	Use big words
strategies	Set topics
	Provide with information they need
	Technology/websites/computer-based programs
Other responses	Video recording/taped answer/DVDs
	Books
	Art
	Coffee/social clubs/groups

## Results

All of the 49 (100%) consenting students completed the questionnaire. The gender distribution of students (2 males, 47 females) was consistent with that of the SLP profession (i.e., few males in comparison to females). The median age of the students was 21 years (range = 18 to 48 years). The majority of students had never had any contact with PWA (n = 39, 79.6%). The remaining students had experienced limited contact with PWA through relatives (n = 2), a friend (n = 1), work experience (n = 1), 1 to 2 hour observation visit (n = 3), volunteer work (n = 2) and paid reception work (n = 1).

The confidence levels reported by participants on the visual analogue scale were not high; median of 29 (IQR =17 to 47) and total range of 2 to 80. The frequency histogram of responses on the confidence rating scale is displayed as Figure 2. The distribution of responses is centred in the lower half of the scale with an exception of 9 (18.4%) respondents who marked near the mid-point of the scale. Only 4 (8.2%) of respondents marked their response above 55 out of 100. The logistic regression indicated that no association existed between degree enrolment (undergraduate versus graduate entry master’s degree) and confidence rating (coefficient = −0.02 [standard error = 0.02], p = 0.22, pseudo r-squared = 0.03, probability > chi2 = 0.22). Similarly, the regression analysis indicated no relationship existed between the age of students and their confidence rating (coefficient = 0.06 [standard error = 0.06], p = 0.310, r-squared = 0.02).

A median of 4 (IQR =2 to 5) strategies were reported by participants in response to the question regarding potential strategies to assist communication between health professional and PWA in clinical settings (range 0 to 9). Thematic analysis revealed the listed strategies could be grouped into four overarching themes; Physical, Verbal Communication, Visual Information and Environmental Changes. The response categories within each theme are listed in Table 1. The most frequent response categories were: use of images (such as pictures, photographs or diagrams) with 31 responses, writing the message (or writing key words) with 25 responses, use of gestures with 20 responses and reduced rate of speech with 18 responses. All other response categories had less than 10 responses each.

Strategies that were reported but considered to be of uncertain benefit or insufficiently described to be able to determine likely suitability are reported in Table 2. Some responses were based around therapy techniques or resources (e.g., colour coding, computer-based therapy) rather than strategies for health professionals to use when communicating with PWA. Others were not described in sufficient detail to be able to be considered suitable or may have been considered potentially beneficial for promoting conversation with PWA in an absolute sense (e.g., coffee groups), but without intent of facilitating communication between a health professional and a PWA where specific information may need to be conveyed. Four students also listed multi-modal communication, nonverbal communication or alternative modes of communication without providing any further specific strategies or information, while an additional five students listed augmentative and alternative communication. At least one of the listed responses (*use big words*) could be considered an unwise strategy choice for assisting communication between health professionals and PWA; although this was not a common phenomenon.

## Discussion

SLP students in the present study did not report high levels of confidence at the prospect of communicating with PWA, despite having completed academic coursework about aphasia. This finding is congruent with the preliminary work by Jagoe and Roseingrave [18] who reported that SLP students may experience apprehension at the prospect of communicating with PWA. This low level of confidence in communicating with PWA may cause anxiety during subsequent aphasia-related clinical placements. Students may benefit from practical training in effective strategies to communicate with PWA before they encounter PWA in clinical settings in order to increase their confidence and promote knowledge of a range of suitable strategies to assist their communication with PWA.

This sample of students identified a range of strategies for facilitating communication with PWA. The most frequent responses listed by the students were: use of images, written information, gesture, and reduced speech rate. These strategies have been previously identified in the research literature as possible techniques for facilitating communication with PWA [25,29-33]. It was interesting that only one of the most popular techniques suggested by the students involved the speaker altering their verbal communication (reduced speech rate), while the other popular techniques (images, written information, gesture) involved supplementing or potentially replacing verbal communication. Despite likely having sufficient knowledge to pass their coursework examinations, the median number of strategies and resources identified by the students for communicating with PWA was four (with some students suggesting no suitable strategies). Interestingly, no student suggested repetition of information or signalling an upcoming change in the topic to the PWA, both of which have been identified as potential strategies for facilitating communication with PWA [33]. This indicates there was room for increased knowledge among the SLP students regarding strategies for assisting communication with PWA.

One potential approach to assist students to build confidence and knowledge of strategies for communicating with PWA includes practical exposure to PWA; such as during communication partner training programs. Communication partner training programs are interventions intended to improve the trainee’s ability to communicate with people who have communication disorders (most commonly aphasia) [34]. Communication partner training programs for aphasia generally incorporate the use of a practical session where trainees practice communicating with a person who has aphasia. Existing empirical evidence suggests that PWA can participate more effectively in conversation with trained partners [8], and that the supported conversation techniques used by trained partners can help to overcome the barriers created by aphasia, enabling re-engagement in health care decisions and everyday life [5,7,10]. Additionally, the study by Jagoe and Roseingrave [17] found that communication partner training programs reduced the apprehension of SLP students when communicating with PWA. This existing literature coupled with findings from the present study suggest use of communication partner training with SLP students is worthy of consideration as an intervention to promote increased confidence and knowledge of effective strategies for communicating with PWA prior to SLP clinical education placements. This might allow students to focus their preparation for placements on more specific clinical skills, and to maximize clinical learning opportunities while working with PWA during their aphasia-related clinical placements.

As SLPs receive specialised training in communication, they were targeted for the purposes of this study. However, there are many other student healthcare populations that do not receive specialised training in communication as a focus of their education programs, but still spend significant amounts of time in their professional roles communicating with PWA. This is particularly true in rehabilitation settings where people who have an acquired brain injury interact closely with health professionals from many disciplines. As a result, future research could investigate the communication confidence and skills of students from other health professions such as physiotherapy, occupational therapy, nursing, and medicine and the effects of communication partner training on these professional populations. This may in turn assist in reducing some of the potential barriers that PWA can experience in the healthcare setting.

### Limitations

In this investigation, the authors considered that the benefits of using a simple and efficient questionnaire design to address the study aims outweighed the potential disadvantages associated with research designs that carry a higher participant burden; which may have in turn resulted in fewer participants and fewer completed data sets [17]. However, several limitations must be considered when interpreting findings from this study due to the nature of the sample and questionnaire. First, self-reported confidence on a visual analogue scale is not the same as actual ability; neither does it provide qualitative information about the nature of any perceived lack of confidence. Students’ actual ability to communicate with PWA may be better (or worse) than their self-reported confidence levels. Similarly, there may be a gap between the knowledge of communication strategies during an open ended recall task and spontaneous use of the techniques during real-life interactions. Direct observation or analyses of video-recordings of students interacting with PWA during their aphasia-related clinical placements (or potentially when completing practical aphasia communication training prior to clinical placements) may provide additional insight into how students apply knowledge from their theoretical coursework in a practical context.

Two important limitations associated with selection bias also exist regarding the nature of the sample. First, the sample was self-selected due to the voluntary nature of participation in questionnaire research. It is plausible that non-responders may have been less (or more) confident or knowledgeable than students who participated in this study. Second, SLP students completing entry level degrees were sampled from a single university. While no association existed between student confidence and their age or whether they were enrolled in an undergraduate or graduate entry masters SLP degree, students completing entry level SLP qualifications that are dissimilar to the program of SLP entry level coursework undertaken at this university may not have provided the same pattern of responses. Nonetheless a description of the coursework undertaken by the students in this investigation has been provided to assist others in determining the validity of any potential extrapolation of findings from this investigation.

## Conclusions

SLP students who had received aphasia related coursework material, but had not yet completed aphasia-related clinical placements, were not confident in their ability to communicate with PWA. Students may benefit from an educational intervention or curriculum modification to incorporate practical training in effective strategies to communicate with PWA, in addition to theoretical coursework, before they encounter PWA in clinical settings. Ensuring students have confidence and knowledge of a range of potential communication strategies to assist in basic communication with PWA might alleviate students’ focus on developing foundation level interpersonal communication during clinical placements and allow the students to instead maximize their learning experiences in more specific clinical domains; such as clinical reasoning.

## Abbreviations

SLP: Speech language pathology; PWA: People with aphasia; CBOS: Competency-based occupational standards; WHO: World health organisation; IQR: Interquartile range.

## Competing interests

The authors declare that they have no competing interests.

## Authors’ contributions

All authors were involved in the study design. EF, AC, KB and JF were involved in data collection. EF and SM completed the data analysis. EF contributed to the principle drafting of the manuscript. EF and SM completed manuscript revisions. All authors were involved in critically appraising the manuscript. All authors read and approved the final manuscript.

## Pre-publication history

The pre-publication history for this paper can be accessed here:

http://www.biomedcentral.com/1472-6920/13/92/prepub
